# Failed in aging? Queering in living with dementia

**DOI:** 10.3389/fsoc.2023.1139271

**Published:** 2023-05-30

**Authors:** Valerie Keller

**Affiliations:** ISEK – Department of Social Anthropology and Cultural Studies, University of Zurich, Zurich, Switzerland

**Keywords:** aging, queering, dementia, self-care, failing, age, norms, ideals

## Abstract

This article explored the ways in which living with dementia brings potentials to queer the concept of “successful aging” and associated notions of being human. Regarding the progressive development of dementia, it can be assumed that people affected, no matter how hard they try, will sooner or later fail to age successfully. They increasingly become a symbol of what is called the “fourth age” and are framed as an essentialized other. Based on statements of people with dementia, it will be examined to what extent the position on the outside enables people affected to abandon societal guiding ideals and undermine hegemonic-dominant notions of aging. It is shown how they develop life-affirming ways of being-in-the-world that run counter to the idea of the rational, autonomous, consistent, active, productive, and healthy human beings.

## 1. Introduction

Aging studies have increasingly borrowed concepts from post-colonial, feminist, crip, and queer studies in recent years. The focus lies on theoretical concepts, such as abject bodies and identities, and structures of exclusion and stigmatization of certain bodies, ways of thinking, and living. Practices of infantilization, the phenomenon of the absent-present body and old-age shame (Rajan-Ranking, 2018), the question of subalternity (Kunow, 2016; Keller, 2022), and various ways of othering people of old age (van Dyk, 2016) are examined. However, the transfer of theoretical concepts is not only helpful in bringing ageist phenomena and related social power relationships to light (van Dyk, 2016) but also proves useful in revealing disobedience, resistant practices, and performances of aging. Against this backdrop, queer potentials of later life are emphasized (Sandberg and Marshall, 2017; Keller, 2022) and forms of mimicry between social age categories are examined (Kunow, 2016; Küpper, 2016). Particular attention is paid to living with dementia, which can be read as an antithesis to “active, productive, and successful” aging because of its connotations with dependence, passivity, and unproductivity (Zimmermann, 2020). For this very reason, Sandberg and Marshall argue, living with dementia exhibits queer potentials: “[I]t is possible to think of dementia in both its cultural and social representations and in its lived experience as a queer experience in old age” (Sandberg and Marshall, 2017, p. 7–8). In light of society's guiding ideal of active and successful aging—which, according to Sandberg and Marshall, is associated with the notion of “heteronormativity, able-bodiedness and able-mindedness” (Sandberg and Marshall, 2017, p. 1)—living with dementia appears to be a failure in aging. However, as soon as we detach the dementia process from dominant framings that define it—such as the loss of one's personality or even one's humanity—forgetting could be rethought (Sandberg and Marshall, 2017, p. 7): Forgetting could then be understood as a release from social obligations (Halberstam, 2011), normative behaviors, or self-imposed barriers (Keller, 2022). This would mean learning to appreciate the liberating and enriching side of failure and recognizing its promising potential. Following Judith Halberstam's arguments in *The Queer Art of Failure* (Halberstam, 2011), as well as initiatives such as *GenderFail*, I will draw attention in this article to failure as a “boundless form of creative potential” (Oakley, 2020, p. 4) and, in relation to the question of “successful aging,” elaborate on how, while living with dementia, dominant notions of aging can be queered in meaningful ways. Failure is understood, in the sense of Be Oakley, “as a tool rather than a social classification” (Oakley, 2020, p. 14) that can prove useful in making visible guiding ideals of aging and elaborating what non-dominant possibilities of pleasurable living may exist in old age.

Yet, in the intentional misunderstanding (Hall, 1973) of the binary categories of success/failure, it should not be misunderstood that living with dementia presents many kinds of challenges and oppositions that individuals affected face in their daily lives in relentless ways. Rather, what follows is about practices that are developed in the interplay of agency and interdependence to seek a coherent way of dealing with challenging situations. It is, therefore, less about resilience, as described by the German cultural sociologist Bröckling (2017, p. 113–139), a personal capacity to adapt to unchangeable circumstances, and more about loopholes and alternatives, small ways in which bodily degenerative processes can be lived in a life-affirming way.

## 2. Queering as a practice of cultural transformation

To begin the endeavor of framing practices in living with dementia as a form of queering, I will first outline what I mean by the practice of queering. According to the Austrian Philosopher Gudrun Perko, queering is first of all an “opened project that radically questions the supposedly natural order of things” (Perko, 2005, p. 27). This statement implies, first, that there is no natural order of things, but, second, that there are things that seem natural to us, and, third, that there are ways to question this seemingly natural order of things. The study of queering is, thus, also always a study of normality and truth. Following Judith Butler's approach, evidence is not considered a criterion for ontology (cf. Gugutzer, 2015, p. 90). In this sense, queer feminist theorists have, among other things, worked out the extent to which supposed truths, such as the binary of gender (male/female) and the associated possibilities of sexual orientations (hetero-/homo-/bisexual), are social constructions. In pointing out the constructed nature of certain behaviors, desires, and meanings, the goal is to uncover the illusion of an innate, natural way of being. According to Butler, we do not use our bodies to express something that is hidden deeply within us—such as an original, inner core, or a true identity (Butler, 1990). Rather, we would reproduce cultural signs in repetitive acts and thereby produce our self-image in the first place. In a performative way, the idea of gender is then copied, but this copy is repeated so often that we ourselves have the feeling of being a gender. According to Butler, this self-deception is made possible by an embodied consciousness into which the repeated movements and expressions are inscribed (Butler, 2002/1997). Embodied consciousness, also called “implicit memory” in psychology (Fuchs, 2010, p. 232), is understood as a bodily memory function that can be called up unconsciously and helps us to repeat complex movement sequences. According to psychiatrist and philosopher Thomas Fuchs, what has once been practiced and sedimented into the body can then be forgotten again and, thus, serves to relieve the burden of attention. Concerning a self-understanding that sediments itself bodily, Fuchs speaks of a *habitus* (Bourdieu, 1993). Following Butler again, this habitus is produced by a repeatedly re-performed idea that inscribes itself into the body through routinization. The collective idea of something then does not have to be remembered and consciously staged every time but is unconsciously recalled in the body memory. This creates a self-deception because it is falsely assumed that the (gender) identity is naturally given (Butler, 2002/1997, p. 309). According to this, the body functions as an institution for creating an illusion: the illusion of having a constant (gendered) self (Butler, 2002/1997, p. 302). In order to maintain this illusion, it could be argued with Foucault (2003/1976), we exclude what runs counter to it, and frame it as a pathological condition. The punishments in the form of stigmatization and exclusion, as well as the societal pressures for therapy that befall the deviant subject, regulate one's own behavior and make us avoid transgressing boundaries of normality. According to Foucault (1976/1975), the potentiality of punishment alone is sufficient to align the subject with the “healthy” norm. Knowing that we can be observed, judged, and excluded by others at any time—whether by exclusion from social groups or inclusion into an institution for the purpose of therapy—we would rather discipline ourselves.

If we assume that a certain form of normality is produced by the illusion of a supposed naturalness that is maintained with the help of self-delusions and (self-)disciplinary mechanisms, then queering could be understood as a practice that makes visible what is mistakenly understood as naturally given and, thus, as unchangeable. Such a practice may appear to some as a weapon to be longed for and to others as a danger to be banished, because making visible the constructed nature of a phenomenon ultimately holds the potential to shift normative boundaries—boundaries that are not infrequently hard-fought. In this definition, the respective practices point beyond questions of identity politics, and, to quote Perko again, aim more generally at “questioning a natural order of things” (Perko, 2005, p. 27). Regarding aging, one could ask which ideas of age(ing) are ritually repeated in *doing age* and essentialized in discourses on aging, and which ideals of age are thereby established as supposedly healthy normality for which to strive. This can then be contrasted with practices of people living with dementia who fail in following the ideals of old age (and therefore being pathologized) but nevertheless do find new, life-affirming, meaningful, and pleasurable ways of being-in-the-world.

At this point, it remains to be said that with the expansion of the term queering, there also comes the danger of diminishing the term's effectiveness for those minorities who have raised it. This is by no means the aim of this article. Rather than an extension of queering, I seek to provide it as a method that is not fixed to defined areas but, in the constant transformation of social structures, must continually address new subjects to fulfill its task: “to make strange, to frustrate, to counteract, to delegitimize, to camp up—(heteronormative) knowledge and institutions” and “to show how they are unstable, fluidic fictions that are the effects of regimes of power/knowledge, which regulate bodies and desires” (cf. King, 2016, p. 53).

## 3. The phenomenon of dementia

The use of the term dementia entails a serious problem: Derived from the Latin term *dementia*, it literally means “being out of one's mind” and is translated as “folly” or “madness, insanity.”^1^ Consequently, dementia is a term that supports devaluing and degrading notions of the condition. People affected, therefore, also use other words to avoid describing themselves in a denigrating way: The members of the self-advocacy group PROMENZ, for example, call themselves “people with forgetfulness.”^2^ Because the word “dementia” is insulting and any use of it hurts her, a member of PROMENZ asks the audience at a public appearance to stop using it.^3^ Using the term “dementia” in this study, I am affirming its use and, thereby, offending people affected. That I use the word nonetheless has the following reason: I am not examining the mental state of certain people, but a sociocultural phenomenon that becomes visible under this term in the first place. I am examining dementia as a phenomenon, which is, indeed, determined by certain behavioral abnormalities shown by particular people, but also by their stigmatization. As the aim of this article was to thwart stigmatizing perceptions of people with dementia, it is to be hoped that I will, in this way at least, make a contribution to change the perception of dementia or render the term obsolete at some point in future—maybe in exchange with the notion of “senility,” a broader concept, which Ward and Price (2016, p. 73) put forward as term that could be reclaimed and, according to Cohen, puts an emphasis not only on biological change but also on an “institutional milieu in which such change is marked, measured, researched and treated” (Cohen, 2006, p. 1).

## 4. Data and methods

This study is based on statements by people with dementia from Switzerland and Austria. The data material goes back to the interdisciplinary project *Self-care in the Face of Dementia. In the Horizon of Spiritual Care and Cultural Studies*, which was conducted at the University of Zurich from 2018 to 2021.^4^ Central sampling methods of the empirical data are the qualitative interview with people affected and participant observations in four different self-help and self-advocacy groups for people with dementia^5^. The analysis of the notes of the discussion groups and interview transcripts was performed by means of a narrative text analysis (Lucius-Hoene and Deppermann, 2002) and the method of open and axial coding according to the procedure of grounded theory (Böhm, 2004, p. 476).

In total, 17 interviews were conducted between August 2018 and May 2019. A single interview lasted between 46 min and almost 3 h and took place either on a walk, on a park bench, at the interviewees' homes, in a café or a restaurant, in a meeting room of a hospital or a conference room of a university. The location for the interview was chosen by the interviewees themselves and was mostly in the immediate neighborhood of their home. The participant observations took place in the period from July 2018 to January 2020 in a total of 24 group sessions, each of which was attended by five to nine people with dementia, a group leader, and me. The sessions have been documented in the form of conversation transcripts.

At the time of data collection, the interview and discussion partners were in an early phase of their dementia development, did not experience any advanced dementia-related impairments, and could all agree to participate in the study in the sense of “informed consent” (Hellström et al., 2007, p. 611). The people interviewed were between 51 and 85 years old; they all speak German, consider themselves heterosexual, male or female, have various socioeconomic backgrounds, and have been living with the diagnosis of dementia for between a few months and 14 years. Following the ethical research principle of “non-harming” (Hopf, 2004, p. 594), the names of the participants speaking are anonymized. The names of people who explicitly expressed their wish to appear with their real names are an exception to this. The reason for this is their conviction that dementia is nothing of which to be ashamed. The request of the people to appear with their correct name shows their emancipatory desire to defy the stigma of dementia. I complied with this request not only to support them in their conviction, but also because the people in question have already presented themselves to the public elsewhere as living with dementia, for example, in the form of dementia activism or as representatives of a public working group of people with dementia. Thus, revealing their names in this study does not expose them to any disadvantages or dangers that they do not already willingly face in their daily lives.^6^

The data material for this study, therefore, comes from people who participate in self-help groups or sociopolitical self-advocacy for people with dementia. They are people who can tell orally what it is like for them to live with dementia. This means that the topic of queering in living with dementia cannot be explored in all its facets based on the data material available. On the one hand, the voices of people living with advanced dementia remain unheeded. On the other hand, the voices of those people who withdraw from social interactions and refrain from involvement in self-help groups or self-advocacy initiatives are missing. In addition, the surveyed group is relatively homogeneous and does not include LGBT^*^ people or people who bring experiences with other illnesses and disabilities.

## 5. The normative order of aging

At the latest since the turn of the millennium, old age has been framed not only as a phase of physical decline and cognitive loss, of social withdrawal and the need for care, but it is also emphasized in a positive way, that old age can be a stage in life characterized by activity, by the realization of dreams or the pleasurable-productive grandparent role, a stage that opens up new possibilities of enjoyment and is marked by an abundance of time, by peace and serenity. This turn to a “new gerontology” (cf. Bülow and Holm, 2016), in which people of old age are not framed in the traditional narrative of decline but are seen as active and productive citizens, is strongly influenced by the concept of “successful aging” elaborated by Rowe and Kahn (1998). According to them, successful agers are healthy, satisfied, active, productive, effective, independent, and self-sufficient people who are “forestalling disease and disability, maintaining physical and mental function, and social engagement” (cf. Katz and Calasanti, 2015, p. 26–27). This fundamental change in the image of old age is perceived positively in critical gerontology, as it offers new opportunities for shaping one's life and social inclusion, but it is also critically emphasized that the image of active aging only relieves those from a negative image of old age who manage to keep fit and healthy (cf. van Dyk, 2016; Zimmermann, 2020). In its focus on individual responsibility to maintain health, it leads to a “narrative of winners and losers” (Rozanova, 2010)—a division between the active and productive *third ager* and the passive and dependent *fourth ager* (Zimmermann and Grebe, 2014)—and overlooks “social relation of power, environmental determinants of health, and the biopolitics of health inequalities” (Katz and Calasanti, 2015, p. 29). Consequently, the notion of successful aging discriminates against people who can no longer be active and productive and, according to McParland et al. (2017), inevitably leads to a second image of old age that diverges from it: an image of frail and dependent aging. According to Zimmermann, this belonging to the so-called fourth age is being hyped up as an existential problem for society. Loss and burden scenarios would be conjured up, in which the social systems would threaten to collapse and metaphors, such as “excess of old population,” “geriatric society,” or “pensioner mountain,” would dominate (Zimmermann, 2020, p. 11). Against this background, people of old age would be referred to their responsibility to the community and, in the case of non-active, non-healthy aging, they would be accused of living at the expense of the younger generation. Thus, a distribution struggle for the supposedly limited financial resources of society would be evoked and the unsuccessful aging would be declared the enemy of a healthy national economy and the horror of relatives (Zimmermann, 2013, p. 101–103). Regarding people of the so-called fourth age, Christine Matter speaks of radical exclusion and an expulsion from the place of the human. A low point of society's discussion on old age is the media's treatment of the “monster” dementia (Matter, 2018, p. 79–92).^7^

Against this backdrop, it seems reasonable to assume that people of old age are subject to constant pressure to remain a productive and consuming force for as long as possible. To remain part of the third age, which is realized in an active, productive lifestyle, the following questions are ostensibly at stake: Can I still support society with community service or consuming power? Can I still manage my everyday life myself, or do I perhaps already need help and, thus, represent a burden for my environment and the social system as a whole? This orientation becomes a challenge particularly in life with dementia: Due to cognitive changes, it gets increasingly difficult to be productively involved in the market economy and relieve family members of additional care work. People with dementia, therefore, repeatedly talk about a painful handover of tasks and responsibilities. The retired physician Bea Gulyn states:

100%?—no, I'd like to, I'd like to, but things come up sometimes, you can no longer count on me 100 %. […] Even with N. [name of her grandchild]. When he hides and I don't know where he is, then I realize, I panic, and then everything gets blurred. […] Many people don't say hello to me anymore because I can no longer do the classic grandma role, but I stand by it. And I wouldn't survive it if something happened to him. As a grandma, well, and I really panic when I don't see him anymore. […] When he walks out the door and there are the cars, I go crazy and immediately run after him, and he doesn't like that, and there's a bit of a distance created.^8^

Following Sandberg and Marshall (2017), being a grandparent is seen as a success by a contemporary society. Accordingly, those who are unable to fulfill the generative task of caring for grandchildren are portrayed as lonely and unhappy old people (Sandberg and Marshall, 2017, p. 20–21). This becomes apparent in the quotation from Ms. Gulyn when she talks about how other people would no longer say hello to her. In the heterosexual matrix of aging, Sandberg and Marshall (2017) argue, the role of grandparent occupies a central place and is presented to us earlier in life as a source of later-life happiness. This heteronormative showcase of an aging future promotes unpaid care work within the family, while, at the same time, constructing a serious void for all those who do not conform to this ideal (Sandberg and Marshall, 2017, p. 3–8). The situation becomes even more difficult for those who are no longer merely unproductive, but who also perceive themselves as an additional burden to others. The former primary school teacher Luisa Marquez tells me about the tiresome situation of her partner always helping her out:

I think it's more difficult—at home, of course—when you can see that it's also difficult for the other person. Because there is always some help […] or something to look for or to do, which […] [VK: So not only for yourself, that now something is misplaced again, but that you are worried/] Yes, that it's also troublesome for the other person. [VK: And have you ever talked about that? Or is it more of a perception of yours?]—(LM cries—recording is stopped).^9^

The idea of being a burden for other people seems to weigh heavily on Ms. Marquez. This is also indicated by the former physics professor Urs Feller: “Well, I suffer a lot from that. I mean, my family, although they actually know it, they just get annoyed with me. Often. And it is also understandable! But I suffer from that too, that's clear.”^10^ The prospect of increasingly becoming a burden on one's environment as dementia progresses can be so frightening that even the option of leaving life prematurely is considered. Some of my interviewees have already registered with *Exit*,^11^ a legal euthanasia organization in Switzerland. The former nurse, Rita Schwager, explains that this registration is a great relief for her:

Exit is always asking [and making sure I don't miss the moment]. For me, that is pure quality of life. […] It allows me to enjoy my life, to soak up the moment. […] And as long as I still have my feelings, everything is still good. But I'm just going a little bit further with this disease now, because for me there is no longer any quality of life if I'm not able—to realize things. […] It gives me high quality of life, that's the most important thing, right? If I had all the time in mind—Jesus Christ!”^12^

What Ms. Schwager would have “in mind” without having the possibility of a self-determined exit from life, she explains to me at another point in the interview: “I want to go with Exit. Because I don't want my children visiting me in a nursing home for another twenty or thirty years.”^13^ In order to be allowed to use the services of *Exit*, there must be a medically certified capacity to judge in evidence. However, to get this proof is constantly at risk of living with dementia, which is why Ms. Schwager remains in close contact with her doctor and the organization *Exit*. According to her, she does not want to miss the moment to leave, but she wants to postpone it as long as possible. With this goal in mind, she has designed an elaborate training program: “I have filled my time with a cognition training program of my own design. On various levels, and I do my training really hard and every day.”^14^ But even without facing the sword of Damocles regarding the possible diagnosis of incapacity to judge, people with dementia are telling about a daily routine in training: There is talk of memory training groups, exercise CDs and exercise books, and Sudokus and crossword puzzles to keep fit in the head.^15^ There is also increased exercise to “keep the body at least a little longer halfway fit”^16^ and perhaps—so it is assumed—to “activate” the brain or “influence the ability of thinking.”^17^ To promote their own health, those affected also change their diet: They give up chocolate and sugar, begin with consuming high-quality nutritional supplements or just try to eat healthier food. Various therapies are attended, and medications are taken, although, according to Ms. Marquez, “at the moment you've already reached the point where you can say you really don't have anything against it.”^18^ But in life with dementia, there is really nothing left to do but go to the specialist and have medication prescribed: “Because that's just the way it is […] you go to the specialist, who do I know who that is, and then you get something prescribed and then you just wait and see.”^19^ Going to the doctor and asking about the possibilities of (medical) therapy seem to be not only a possible first reaction, but the only possible one at all, which is socially accepted and encouraged. As a pathological condition, as dementia is framed in its deviation from a normal aging process, it calls for medicalization, therapy, and training. It is, therefore, not surprising that, according to Alzheimer Switzerland, the aim of drug treatment of dementia is “to maintain mental performance and everyday functions for an extended period of time” or “to delay the decline in mental performance and to reduce dementia-related behavioral disorders.”^20^ Against this backdrop, it becomes clear how images of old age and the normative order of aging affect the living with dementia: The activity, independence, and productivity demanded forces people with dementia to take a path that, given a progressive dementia process, can only end in failure. What Zimmermann (2020, p. 31–32) articulated in relation to active aging applies even more forcefully to people with dementia: Even the best agers would eventually surrender to what they have rejected. In living with dementia, failing is inevitable.

## 6. Letting go

Considering the situation that failure in aging is so severe that the option to step out of life in a self-determined way becomes a relief, the question arises: “Whose lives are regarded as lives worth saving and whose are not?” (Butler, 2010, p. 38). The statements made by Ms. Schwager about her daily training clearly show her effort to preserve a certain form of life. However, she attributes no quality of life to another form of life, which she associates with an advanced stage of dementia: She would rather die than experience this. According to Butler, “life can be accorded a value only on the condition that it is perceivable as a life, but it is only on the condition of certain embedded evaluative structures that a life becomes perceivable at all” (Butler, 2010, p. 51). If a life is perceived as a life only when it is apprehended by certain entities of valorization, this leads us to the question with which entities of valorization Ms. Schwager judges the quality of her anticipated future life.

Ms. Schwager's arguments about when she herself considers her life no longer worth living derive from moments of dependency, the anticipated loss of being able to take on the previous role as mother and the fear of no longer being able to perceive anything. As she states, she would need professional support to cope with everyday tasks and her children would have to come and visit her many times, although she assumes that, at some point, she will no longer be able to “realize” anything herself. Thus, she points not only to a decline in cognitive thinking but also to loss of bodily abilities—specifically, the ability to feel anything: “[A]s long as I still have my feelings, everything is still good. But I'm just going a little bit further with this disease now, because for me there is no longer any quality of life if I'm not able—to realize things.” In her arguments about when her life is no longer worth living for her, she, thus, combines the guiding ideals of a neoliberal society—activity, productivity, and performance—with the idea of the modern subject (cf. Eggmann, 2022): a rational, autonomous, consistent, and self-reflexive being who understands their mind as an essential core and uses their body as a vessel that stops feeling when sensations can no longer be processed by cognition (Fuchs, 2010, p. 231). Following Ms. Schwager, life with advanced dementia is, therefore, questioned as a life worth living because it fails to live up to the idea of a subject based on ideals of the Enlightenment—an idea of a subject that, according to educationalist Brinkmann (2008), is the foundation for the concept of successful aging. According to his argument, the notion of the human beings as an active, autonomous, cognitive-rational ego identity has led to a power relation that manifests itself in the compulsion to “adopt autonomy productively and successfully” (Brinkmann, 2008, p. 284). The Enlightenment, he argues, has turned into its opposite, as “the demand for autonomy becomes the compulsion of modern techniques of individualization and neoliberal strategies of governance” (Brinkmann, 2008, p. 238). Against this backdrop, a life characterized by vulnerability, dependency, and reduced cognitive capacity is framed not only as a failure in successful aging but as a loss of being fully human. This tendency of dehumanizing people with advanced dementia is also evident in public media discourses. A study by Grebe et al. (2013), in which 250 articles from German newspapers, magazines, and advisory literature in the period between 1990 and 2011 were examined regarding the metaphorical construction of dementia, shows that dementia is described with metaphors that refer to absence, loss and regression. Dementia is repeatedly described as a journey into a fundamentally different reality (“the journey into the land of forgetfulness” or “the way into no-man's land”), in which, once there, the mind is no longer accessible to other people. All that is left behind is a body that, henceforth, exists as an “empty shell” (Grebe, 2019, p. 210). People with dementia, Grebe et al. (2013) elaborate, are metaphorically described as “phantoms,” who, with the ongoing loss of their memory, lose not only their connection to reality but also their personality and selfhood.

What is equally evident in Ms. Schwager's anticipated vision of the future and media representations of dementia is an essentialization of the modern subject: The natural order of being human is found in an autonomous, active subject, which forms a coherent, rational unit. Those who fail in living up to this ideal are, therefore, questioned not only about aging successfully but also being fully human. In relation to this, people affected speak of multiple experiences of stigmatization and exclusion, which make it difficult for them to maintain previous structures of meaning in their lives (Keller, 2022). They are denied the need to participate in society, Ms. Schwager explains to me, “simply because—somehow it has been forgotten that this could be a need at all. Because one simply doesn't expect anything anymore from an Alzheimer's patient.”^21^ However, when positioned on the outside and deprived of the possibility of renormalization, living with dementia also offers the potential to question essentialized notions of successful aging and associated notions of a life worth living. It offers the possibility of generating new ideas about what aging can look like apart from trying to hold on to rationality, autonomy, consistency, activity, productivity, and health. The awareness of having failed, therefore, makes my interview partner and former pastor Franz Inauen reconsider his own ideals:

Having to let go, being able to let go, that's my next topic, my weekly topic. [VK: Letting go of fixed ideas of how it should be, or letting go of/] A little bit of everything. Of values, ideas, and especially ideals, yes. […] [VK: You write [in your poetry collection] about new horizons appearing.] But they follow another direction. Until now, it was in the sense of achievement, values and so on. As much as possible in a short time. I must let go of that now.^22^

As soon as Mr. Inauen begins to detach himself from achievement and productive optimization as ideals to strive for and accept unknown turns in life, new horizons open up for him—horizons that would lead into a different direction than the striving for performance would. Repeated failure consequently makes individuals affected say goodbye to previous ideals. Here, it is interesting to note in Mr. Inauen's statement that he attaches two verbs to let go: “Having to let go, being able to let go.” On the one hand, letting go of ideals imposes itself on him in living with dementia; on the other hand, letting go also requires ability, which, in turn, makes it an intentional action. This is also described by Ms. Gulyn when she talks about things that she can no longer do, but which she also no longer wants to do: “I have visibly disengaged from society, I also see it as a weakness, that I can't perform anymore, but also see that I don't want to perform, in a lot of things, nah.”^23^Against this backdrop, Ms. Pototschnigg establishes a practice she calls “expansion.” She cannot change dementia and its effects, so she prefers to focus on other things. In order not to be limited by things that no longer work, she tries to reduce such limitations that she has imposed on herself:

I also expand quite a bit into music. I come from hard rock, I like that, yes? […] I've only added a little folk music or reggae music […] over the years, but was somehow convinced, as it went to 60, that it is enough. That's enough, if you have four or five different directions, that's enough. Then you can get through well. […] Today, amazingly, you might find me at piano concerts, you might find me at the concert hall, at the opera, the spectrum has become larger, yes? […] And I think that must also have happened in my head, that this wideness takes place, or that I allow it to myself. […] I really feel the width, I hardly went on vacation because I was afraid of flying. […] But that's no longer the case today, it's gone. Expanses that I get to know, of which I can say, how can this happen, are these fears in my head? Probably, because they are no longer there for me today. And […] I'm afraid of dogs, always have been. […] Today, where I live, quite a lot of neighbors have dogs, I often say: ‘Can I walk next to your dog for a while? I'm so terribly afraid of dogs.' […] that's what I mean by expanding. To try out! […] I expand with what I was used to and thought, that's me. I realize that I can do even more! […] I realize that I have limited myself so much in the image of myself. There is so much more possible! I think that's so beautiful! I mean, I think the forgetfulness is less beautiful, […] but it's not like I say, ‘Boo, I have the disease now, so much is being taken away from me!' What I mean to say is, I'm also given a lot in terms of new opportunities, new things, new interests, which I accept very gratefully and […] it would have been a shame if I'd always stayed the Angela and never had the opportunity to experience that!^24^

Ms. Pototschnigg allows herself “expansion.” Fixed ideas of what she was, fears that she had “unconsciously” acquired, and blockades for which she was responsible had limited her in many ways. By confronting the self-imposed restrictions, she opens new possibilities for herself. One of the “most serious things” she allows today, she tells me, is pausing a while and having a little chat when meeting other people—be it at the neighbor's garden fence or at the bus stop among strangers:^25^

In the past, it was said that you don't talk to strangers, I would have turned around and thought: “What does he or she want from me?!” […] Today I really like that, because it happens more often that the ladies are of course my age, who say: “Don't we have nice weather today?”—“Yes, you're absolutely right!”—“Yes, we can hope that it will still be like this tomorrow!” And I think to myself: “Great! Right?” And I have a good time, I consider it an absolute enrichment. […] Sometimes other old ladies arrive on the bus, and then the four of us sit there and laugh about something, chat quite well and have it quite fine (laughs).^26^

Striking up a conversation with complete strangers on the street is something she used to think one doesn't do. Even more, she would have perceived such an incident as a disturbance in her everyday life. Knowing that it's not quite appropriate, she is still a bit cautious but “the more it happens, the more I get the courage to approach someone myself (laughs). […] I realize what joy it can be.”^27^ What Ms. Pototschnigg has been enjoying lately is not an autonomous, goal-oriented life but unexpected gatherings and shared moments with other people. These and other new qualities in her life have been made possible by turning away from a fixed idea of herself, of what she is and always has been. These ideas, she realizes, are less a result of a true self but are much shaped by social interaction and societal ideas of what one should be, which she now perceives as limiting the variety of possible forms of existing. In the practice of expansion, she begins to behave in a way that contradicts her former self-image and overcomes fears related to her own vulnerability by walking beside dogs and using airplanes. It is a way of living, which accepts inconsistency, relationality, and vulnerability as part of a human's life.

Vulnerability and finitude, as former logistics specialist Brigitte Feldmann^28^ makes clear to me, too, accompany people throughout their lives. When asked about her everyday dealings with dementia, she starts telling me episodes from the life of her father, who recently passed away. It is a miracle that he lived as long as he did, she remarks, because he had already narrowly escaped death at least twice. The planning of her own funeral comes into play shortly afterward when Ms. Feldmann is talking about a song she wants to play, which was already played at the funeral of a former partner who died much too early in a tragic accident. In her narrative, the experience of vulnerability and finitude appears almost as a guiding principle of her life and, in relation to her current situation, is framed less as a tragedy and more as a basic condition of being human. Death has already caught up with others and will eventually reach her as well. Similar sentiments are expressed by Ms. Gulyn^29^, who shows me her mother's grave before the interview and, during the walk in the cemetery, leads me to the entrance gate where it is written in stone: “We were what you are, you will become what we are.” She finds this saying very true, but most people were blind to it. She would rather die than blindly go with the flow of society, she explains to me. After all, “day and night […] are also part of life, aren't they? At some point it gets dark! (laughs).”^30^ Later in the interview, she gives me an indication of the extent to which Ms. Gulyn does not participate in society and, therefore, prefers to risk death: She does not take any medication; this life “behind glass” is nothing for her. Even as a physician, she preferred to find out what was wrong with people rather than prescribing medication. She now applies this approach to herself as well: What she lacks above all, she says, is permission to “wither.” Nature would demonstrate this process to us every autumn, and we would enjoy the falling leaves. But there is a lack of social acceptance for similar processes in humans. People were blinded in this respect. Only their performance was seen:

For me, growing old is not a disease, pathological wise, but it is a development, like spring, summer, autumn and winter we have toddler, teenager, adult and now I'm in the fourth stage, old age, right? And these are changes and the leaves are wilting, and I don't see at all, we are part of nature, why, even the cars are breaking down and getting old, no? I don't understand why this rhythm is seen as pathological.^31^

The possibility of bypassing a societal “ban on withering” is something that Ms. Pototschnigg would also like to see: “It's actually a toil for both—at the beginning and at the end and I don't see—I see it as a circle, I don't see it as something quite terrible, but I say it's actually a […] well, an intense leaving of this world.”^32^ Ms. Pototschnigg and Ms. Gulyn, like Ms. Feldmann, locate themselves in a “natural cycle” that integrates finitude from the beginning, and in which fragility and vulnerability can be an intense experience. They accuse society, however, of only being able to grasp achievement and for overlooking the beauty in withering.

By declaring expanding and withering as forms of human existence, even naming them as an intense form of being-in-the-world, my interviewees expand the idea of what it means to be human. By raising the argument of a “natural condition,” they do not detach the notion of human existence from essentializing framings, but they expand the dominant idea of being human by integrating the alleged opposite: Human beings can not only be autonomous, cognitively agile and consistent, but also vulnerable, embodied, and inconsistent. In the inclusion of the outcast from the framework, they dissolve clear ideas of what it means to be human.

## 7. New horizons

Another pleasurable practice comes to the fore when moving away from the claim of successful aging and allowing oneself an expansion into unknown fields of being-in-the-world: lingering in time. It is a practice that moves away from the claim of a productive optimization of time and offers opportunities to simply linger in a situation, thought, or feeling for as long as the lived moment requires it. It is not just about taking more time because he has slowed down; Mr. Maurer, Swiss representative of the European Working Group of People with Dementia, tells me. It goes beyond that. For him, allowing himself more time also means taking as much time as he needs so that he is satisfied both during the activity and with the result of the activity: “I don't regret the time used, I see that it is used positively in the sense of: I am satisfied with the result of my work and I am much less stressed during my work, respectively, much more relaxed and also more satisfied with what I am doing.”^33^ His use of time leads to a completely new quality in his life. In the past, he was “rushed” from one deadline to the next, and he was “breathless on the move,” whereby satisfaction did not arise during the execution of the work and not necessarily with the result of the work—he was satisfied when he met a deadline. Today, he can enjoy many activities because he allows himself as much time as necessary to experience the quality of the moment.^34^ In the practice of lingering and the pleasurable indulgence of a moment, it is possible to lose reference to time. It can happen “that time somehow subjectively changes in length,” Ms. Marquez explains to me, and that she suddenly finds herself having spent hours rearranging a box. She also describes her approach to time as “maybe a little less coordinated.”^35^ But you just have to learn to “enjoy the moment, no matter what, […] even if the world is crashing,”^36^ Ms. Gulyn explains.

In the practice of lingering, the experience of time can change. Time can freeze or expand, and one can temporarily fall out of a shared experience of time. In letting go of certain ideals of productivity, it becomes possible to perform an action or live a moment without adopting a time-constructing perspective, thus focusing on the present relationship to the circumstances and events that arise. This is seen as a new quality in life, because the present is not timed and is not guiding to a guillotine-like deadline but leaves space to live the specific quality of the moment. What is described here by people with dementia are practices that affirmatively resist the normative organization of time. A queer temporality is developed that resists what Elizabeth Freeman calls chrononormativity: “the use of time to organize individual human bodies toward maximum productivity” (Freeman, 2010, p. 3; cf. King, 2022, p. 3). It is a temporality in which life can be experienced in an illegitimate-unproductive but intensely pleasurable way.

This leads me to a final practice that people living with dementia opened up to me: It is a bodily practice of being-in-the-world that I call here “embodying relationality”—a practice that resists the ideal of hypercognitive beings and enables a new kind of bodily embedding in a physical and social environment. Ms. Gulyn no longer participates in society through rational discussions or consumption of cultural activities that are designed for cognitively agile individuals. She does not really feel like going to the movies anymore; she tells me in the interview. Even a TV thriller she watches for no more than 15 min, and this is only to experience the atmosphere arising from sitting comfortably in front of the TV together with her partner.^37^ Her new cinema, therefore, consists much more in activating all her senses and getting involved in a bodily way in social events:

So, for me, going out, I'm a sensual person and I must smell, taste, get the air and feel the people. […] I, for example, like the fact of having a lot of foreigners in the subway, I always sit down with them, I think that's nice. I like the idea that I don't need to go abroad anymore. […] Those are my film experiences, right? Or someone does spill beer, in the past it was not like this, but now they even file a complaint—you're not allowed, you're not allowed… And then, I just ate something, but that was a long time ago, (laughs) and had a napkin in my hand and then I saw the horrified looks of the people, how the beer was on the floor, and then I just gave him the napkin and he knelt down and wiped it away, right. And the mood switches immediately. This is cinema for me. Cinema live.”^38^

Ms. Gulyn embarks on journeys through different cultures by sitting in the subway next to those people who seem foreign to her and, therefore, gives her the feeling of being part of a larger world. By creating physical proximity in her choice of seats, she can establish a bodily relationship with other people, she can “feel” them, to use Ms. Gulyn's words. However, she does not describe the bodily relationship with other people exclusively in a passive way but also tells of active interventions in social events: Not only does she smell the city, observe glances and feel moods, but she also enters into a relationship with them and causing an effect on them. Her offering a napkin, for example, can significantly change the mood of the social group. In body-related interaction, Ms. Gulyn integrates herself into social events and, thus, becomes part of the living world surrounding her.

The bodily interaction with and enjoyment of a physical environment is also of great importance for Ms. Feldmann. She talks to me about the experience of having climbed into the nearby stream overflowing with water after a rainy day and having slowly walked upstream, feeling the weight and power of the flowing water on her body.^39^

And I mean if I get in there, I can really go in there. And then there's this bridge that I stop under, and there's this arch, such a beautiful arch, you can cross it with five tons and nothing happens! That's really such a beautiful old bridge, I am so fascinated by that. Things like that, or nature, fascinate me insanely. There I really—huh—feel alive!”^40^

In the intertwining of her body with the physical world surrounding it, Ms. Feldmann feels that she is alive. She feels the pressure and temperature of the water on her body, she feels the weight of the truck on the bridge and the strength of the arch that supports the weight. She also hears, sees, and smells the nature around her and is fascinated by it. Ms. Feldmann and Ms. Gulyn are, thus, not concerned precisely with entering the “order of the instrumental, of making and doing” as an “active, autonomous, cognitive-rational ego-identity.” That is how Brinkmann (2008, p. 248–249) describes a modern conception of humans, from which the notion of old age as a phase of “health, autonomy, and activity” (Brinkmann, 2008, p. 239) feeds. They both feel life much more in “body-related reflection” (Brinkmann, 2008, 249). Lingering in time and embodying relationality can, therefore, be seen as practices questioning the hegemonial idea of the *cognonormative subject* (King, 2016, p. 59). By interacting with their (social) environment in a body-related way, they bring up the instability of the temporal structure and the possibility of *embodied selfhood* (Fuchs, 2010; Kontos et al., 2018).

## 8. Conclusion

Regarding the implications of dependence, unproductivity, and cognitive impairment, people with dementia are distinct from what is understood by successful aging in modern Western societies. Autonomy, productivity, and able-mindedness are not only presented as supposedly healthy, normal attributes for which to strive, they also refer in an essential way to what it means to be human in the discourses on aging. The notion of successful aging is fed by the idea of the modern subject—a rational, capable, consistent, and autonomous being who uses their attributes to contribute to society in productive ways. Against this backdrop, living with dementia appears not only as a failure to age successfully but also as a failure to be fully human. As dementia progresses, people affected are increasingly framed as essential others and relegated to the place of the abject and monstrous (Bülow and Holm, 2016). The dehumanization of people with dementia can eventually lead to the assumption that a life with advanced dementia is no longer a life worth protecting.

This framing leaves people with dementia with only one way to deal with physical degenerative processes that are supported by society: The effects of dementia must be treated as best they can, and the remaining abilities must be activated as long as possible. In view of the progressive development of dementia, this path eventually must lead to failure. However, this failure can be interpreted in different ways. Either the failure is seen as increasingly severe until it runs counter to what, in hegemonial terms, is understood as “human,” or the failure to age successfully is seen as chance and taken as an opportunity to critically reflect on essentialized ideals of successful aging. It is precisely in its framing as the “other” that living with dementia, thus, offers queer potentials. After all, the essential other, according to Butler, is never to be understood entirely outside of norms. “[T]hey are other and within” (cf. King, 2016, p. 60), from where the other can impact normative frameworks in their otherness. The non-human always refers to the idea of the human, which can be questioned by life-affirming practices of the supposedly “not quite human beings.” Although people with dementia report painful experiences of exclusion and stigmatization, the position outside of normality also provides a foundation to abandon the very hegemonic ideals that are responsible for their exclusion. In setting aside ideals of autonomy, rationality, and productivity, it becomes possible for people with dementia to perceive instability, vulnerability, and bodily relationality as intensive forms of being. The consistency of the modern subject is dissolved in pleasurable expansion, health brimming with activity, and productivity is challenged with life-affirming practices of withering, and the idea of ratio and cogito as the foundation of humanity is countered with bodily practices of being-in-the-world. By giving quality of life to the very qualities that put people with dementia at risk of being excluded from the place of the human, boundaries between the human and the nonhuman become porous. This opens up the possibility of rethinking what we mean by being human.^41^

Losses, vulnerability, and dependence on other people may not disappear as dementia proceeds. Instead, it could be supposed that they become more and more urgent. Although the present study does not include people living with advanced dementia and, therefore, leaves a gap that can, at this point, only be filled with assumptions, it could be argued that practices of temporal and personal fluidity, responsiveness to bodily degenerative processes, and embodied relationality are still important to establish a personal quality of life in later stages of dementia. Whether the aforementioned practices can then still be understood as practices of queering depends on our understanding of queering. Does it have to be a cognitive, rational process in which normative structures are being deconstructed in a conscious way? If so, this would imply that queering can only be practiced by those who fulfill *cognonormativity* (cf. King, 2016, p. 59) to some degree. With that in mind, I return to Butler's thinking again at the very end: Perhaps it does not matter whether people with advanced dementia have the required cognitive abilities to recognize normative structures—they are always within them and act upon them in being-in-the-world. Without normative ideas of healthy, successful aging, the phenomenon of dementia may not exist.

The present study is based on a sample of German-speaking people in the early stages of their dementia development who participate in self-help groups or sociopolitical self-advocacy for people with dementia. The findings, therefore, do not claim a representative character but provide insights into possibilities of living with dementia which opposes and subvert dominant perceptions of successful aging. However, further research on queer potentials in living with dementia remains to be done—including voices of people in advanced stages of dementia development, those of people living with dementia who withdraw from social interactions, LGBT^*^ people with dementia, and people affected who bring experiences with other illnesses and disabilities.

## Data availability statement

The raw data supporting the conclusions of this article will be made available by the authors, without undue reservation.

## Ethics statement

Ethical review and approval was not required for the study on human participants in accordance with the local legislation and institutional requirements. The patients/participants provided their written informed consent to participate in this study. Written informed consent was obtained from the individual(s) for the publication of any potentially identifiable images or data included in this article.

## Author contributions

VK is the exclusive author of this article. She has collected the data material in the context of the third-party funded project “Self-care in dementia in the horizon of spiritual care and cultural studies” from 2018 to 2021 at the University of Zurich, Switzerland.

## Footnotes

^1^Online Etymology Dictionary: https://www.etymonline.com/word/dementia (Accessed: March 24, 2023).

^2^PROMENZ: https://www.promenz.at/zweck (Accessed: September 15, 2020).

^3^Participant Observation on August 11, 2018, at Demenz Meet in Zurich, Switzerland.

^4^Cf. ISEK, University of Zurich: www.isek.uzh.ch/de/populärekulturen/forschung/projekte/drittmittelprojekte/selbstsorgedemenz.html (Accessed: December 14, 2022).

^5^The groups *Labyrinth* in Olten, *PROMENZ* in Vienna, *Impuls Alzheimer* in Berne, and the *Discussion Group for Younger People with Dementia* in Lucerne were visited from July 2018 to January 2020.

^6^Code of Ethics of the German Sociological Association: Persons who are included in studies as observers or in any other way must not be exposed to any disadvantages or dangers as a result of the research. The persons concerned are to be informed about all risks that exceed the measure of what is usual in everyday life (cf. Hopf, 2004, p. 594).

^7^However, although people are increasingly affected by discrimination if they fail in active and productive aging, it would be shortsighted to assume that the idea of third agers is entirely unaffected by ageism. Van Dyk shows what new problematic demands this third age encounters: Due to a glorification of the patient, loyal younger age, they would be subject to the indirect pressure of care and charity work. The young old were ostensibly valued because of their “primitive innocence” (van Dyk, 2016, p. 114), which, coupled with an active lifestyle, enables them to do care work. In contrast to the idea of *midagers*, who were seen to be “closer to culture” through hard work and rational participation in competition, people of old age were seen to be “closer to nature” through their lifetime proximity to death. While the fourth age is associated with illness, vulnerability, and dependence and, therefore, refers to the other, the third age is characterized by experience, loyalty, trustworthiness, patience, reduced desire for competition, and an increased sense of charity and, thus, represents a selfless reversal of such a society, which is focused on output and success. Although there is a lot of appreciation for the third age, it contrasts sharply with the values of flexible capitalism—flexibility, mobility, creativity, and risk-taking—and is, thus, associated with devaluing. In the form of the supposedly positive “affirmative othering,” an ambiguous process that goes hand in hand with the fascination of the oppressed, third-ager would be pushed into the backyard of capitalist society and tasked with taking on unpaid care work and emotional labor (van Dyk, 2016, p. 109–114).

^8^Interview with Beatrix Gulyn on September 9, 2018, in Vienna, Austria.

^9^Interview with Luisa Marquez on September 6, 2018, in Vienna, Austria.

^10^Interview with Urs Feller on January 23, 2019, in Zurich, Switzerland.

^11^Exit Switzerland: https://www.exit.ch/en/ (Accessed: December 21, 2022).

^12^Interview with Rita Schwager on August 17, 2018, in Immensee, Switzerland.

^13^Ibid.

^14^Ibid.

^15^Self-help group *Labyrinth* in Olten, Switzerland, July 2018.

^16^Interview with Luisa Marquez on September 6, 2018, in Vienna, Austria.

^17^Interview with Thomas C. Maurer on April 25, 2019, in Berne, Switzerland.

^18^Interview with Luisa Marquez on September 6, 2018, in Vienna, Austria.

^19^Ibid.

^20^Alzheimer Switzerland, medical therapy in living with dementia: https://www.alzheimer-schweiz.ch/de/ueber-demenz/beitrag/medikamentoese-therapie-bei-demenz/ (Accessed: November 23, 2020).

^21^Interview with Rita Schwager on August 17, 2018, in Immensee, Switzerland.

^22^Interview with Franz Inauen on August 8, 2018, in Lucerne, Switzerland.

^23^Interview with Beatrix Gulyn on September 5, 2018, in Vienna, Austria.

^24^Interview with Angela Pototschnigg on September 4, 2019, in Vienna, Austria.

^25^Ibid.

^26^Ibid.

^27^Ibid.

^28^Interview with Brigitte Feldmann on October 23, 2018, in Kerzers, Switzerland.

^29^Interview with Beatrix Gulyn on September 5, 2018, in Vienna, Austria.

^30^Ibid.

^31^Ibid.

^32^Interview with Angela Pototschnigg on September 4, 2019, in Vienna, Austria.

^33^Interview with Thomas C. Maurer on April 25, 2019, in Berne, Switzerland.

^34^Ibid.

^35^Interview with Luisa Marquez on May 7, 2019, in Vienna, Austria.

^36^Interview with Beatrix Gulyn on September 5, 2018, in Vienna, Austria.

^37^Ibid.

^38^Ibid.

^39^Participant observation at Brigitte Feldmann's home in Kerzers, Switzerland, January 23, 2019.

^40^Interview with Brigitte Feldmann on October 23, 2018, in Kerzers, Switzerland.

^41^According to Butler, enduring losses and vulnerability and integrating them into understandings of humanity might even be something that would make the concept of the human more humane: “I think that an entirely different politics would emerge if a community could learn to abide with its losses and its vulnerability. It would know better what its ties to other people are. It would know how radically dependent it is on its interrelationship with others. I think that would lead in fact to a multi-lateral international understanding of justice, or at least it could. I think we would be able to understand something about the general state of fragility and physical vulnerability that people—as human—live in. Our increased attunement to that could only make us more humane” (Butler, cf. Stauffer and Butler, 2003, p. 102).
